# The N-P-K soil nutrient balance of Portuguese cropland in the 1950s: The transition from organic to chemical fertilization

**DOI:** 10.1038/s41598-017-08118-3

**Published:** 2017-08-14

**Authors:** Miguel Carmo, Roberto García-Ruiz, Maria Isabel Ferreira, Tiago Domingos

**Affiliations:** 1 Linking Landscape, Environment, Agriculture and Food (LEAF), Instituto Superior de Agronomia, Universidade de Lisboa, Lisbon, Portugal; 2 CEAOAO & CEACTierra, Department of Animal Biology, Vegetal Biology and Ecology, Universidad de Jaén, Jaén, Spain; 3 MARETEC, Environment and Energy, Department of Mechanical Engineering, Instituto Superior Técnico, Universidade de Lisboa, Lisbon, Portugal

## Abstract

Agricultural nutrient balances have been receiving increasing attention in both historical and nutrient management research. The main objectives of this study were to further develop balance methodologies and to carry out a comprehensive assessment of the functioning and nutrient cycling of 1950s agroecosystems in Portugal. Additionally, the main implications for the history of agriculture in Portugal were discussed from the standpoint of soil fertility. We used a mass balance approach that comprises virtually all nitrogen (N), phosphorus (P) and potassium (K) inputs and outputs from cropland topsoil for average conditions in the period 1951–56. We found a consistent deficit in N, both for nationwide (−2.1 kg.ha^−1^.yr^−1^) and arable crops (−1.6 kg.ha^−1^.yr^−1^) estimates, that was rectified in the turn to the 1960 decade. P and K were, in contrast, accumulating in the soil (4.2–4.6 kg.ha^−1^.yr^−1^ and 1.0–3.0 kg.ha^−1^.yr^−1^, respectively). We observed that the 1950s is the very moment of inflection from an agriculture fertilized predominantly through reused N in biomass (livestock excretions plus marine, plant and human waste sources) to one where chemical fertilizers prevailed. It is suggested that N deficiency played an important role in this transition.

## Introduction

The stability of cultivation and the persistent growth and specialization of agricultural production in Europe during the modern times and until the late 19^th^ century depended, after centuries of continuous cropping, on the adequate replenishment of soil nutrients^1–4^. The subsequent transition from that millennial solar based agriculture towards an industrial one, fueled by synthetic fertilizer and motor machinery can only be firmly understood, as in the case of previous transformations, through a careful analysis of soil nutrient cycling. Nitrogen’s global history by Smil^5^ is particularly meaningful in this regard as the evolution of yields and agroecosystem configuration are discussed from the standpoint of biophysical and agricultural limitations to N recycling. The adoption of a perspective from agronomy, without denying the importance of many other perspectives on agricultural transformation, as stressed by Overton^6^, introduces new relations that were largely omitted by historical studies on Portuguese agriculture. Furthermore, the combination of quantitative tools for nutrient cycling assessment with the analysis of farming systems transformation has been advocated as a fruitful way to open up the range of historical review and thinking^7^.

“The legumes and especially the lupin, which is the richest, are true nitrogen factories that are available to everyone and do not require workers or machinery, or the need to ensure against strikes”^8^. In this passage, written in the 1920s, in which Portugal moved from the brief First Republic to the fascist-type regime*, Estado Novo*, legumes were in the author’s opinion a better way to provide nitrogen to the soil in comparison to that of a less controllable industrial production. The debate over crops’ nutrients supply went through the entire Portuguese 20th century gaining and losing its relevance in face of major rural and agricultural transformations. Since the late 19th century Portuguese agronomists began to measure nutrient inputs due to manure, legumes, and other biomass paths, including the novel chemical fertilizers, and soon they started measuring as well farm outputs, harvests and straw, from which the intensity of fertilization should be defined^9^. Fifty years later, a nitrogen, phosphorus, and potassium (N-P-K) balance of Portugal arable crops was proposed for the agricultural year of 1952/53^10^ that, besides providing data to our study period from a simple but coeval model, left a challenge for future researchers: “It is materially impossible to make such a ‘balance’ – rigorous and nationwide - without the precise knowledge of the nature, quantities, and composition of all products born and taken from the fertilized ground, including therefore the so called crops, but also the weeded and cut off plants.”

In the last three decades, nutrient balances in a historical perspective have gained momentum as a way of reaching sounder conclusions on agroecosystems’ history and functioning. They combine past information on weather and soil, cropping pattern and yields, main management practices, among other data, with current predictive models of nutrient cycling in soil. Yet, most applied models lack one or more important processes, e.g., N leaching or soil weathering, and most efforts have concentrated on the N balance, disregarding the importance of P and K^11^. More recently, N-P-K analysis became the basis of several historical nutrient balances studies embracing most input and output processes. These have been applied at spatial scales from villages or parishes^11–13^ to countries^14^ or even to the globe^15^. In general, the agroecosystem is analyzed as a whole, comprising all land uses in a given region, but some studies have focused on just one crop^16^ or crop rotation^17^.

Each of the nutrients considered has its own natural and industrial history, as well as specific soil geochemistry. N has a long modern history^5, 18^ and is still a significant topic^15, 19, 20^. The attention devoted to P nowadays results largely from its restricted global reserves and because it keeps offering key missing links in agricultural history^21–23^. K became a “forgotten nutrient”^24^ and is considered not as important as N and P, even though it plays a major role in plants physiology^25^. K reserves appear to be sufficient for hundreds of years but the profitability on marginal soils will depend increasingly on its efficient use^26^.

In this study, we examine the soil fertility of Portugal’s cropland in the 1951–56 period using a nutrient balance approach that seeks to cover all the N-P-K inputs and outputs from the topsoil. The 1951–56 years are very rich in agricultural data and place the analysis at the beginning of the broad agrarian transformation of the 1950s and 1960s in Portugal^27–29^. During the 1950s, Portugal achieved the largest cropland area ever (more than 5.6 million ha, 64% of the country area), which had been increasing since the end of the 19th century from about 3 million ha. The retreat started in the turn to the 1960s and continued until today^28, 30^. This same pattern was observed in the wheat area^31^.

The main purposes of this study were to (1) develop a full nutrient balance model, (2) present a comprehensive assessment of the functioning and nutrient cycling of past agroecosystems, and (3) discuss the main implications for the history of agriculture and technology in Portugal. We first present the study area and the N-P-K mass balance model, including primary data and methods. The results and discussion are organized in three main parts: (1) the nationwide level, which accounts for all cropland uses, the disaggregated balances for (2) the arable crops and (3) the wheat crops, followed by an overall analysis of N-P-K results in these three levels. We include also a sensitivity analysis of results.

## Materials and Methods

### N-P-K balance model

**Figure 1:**
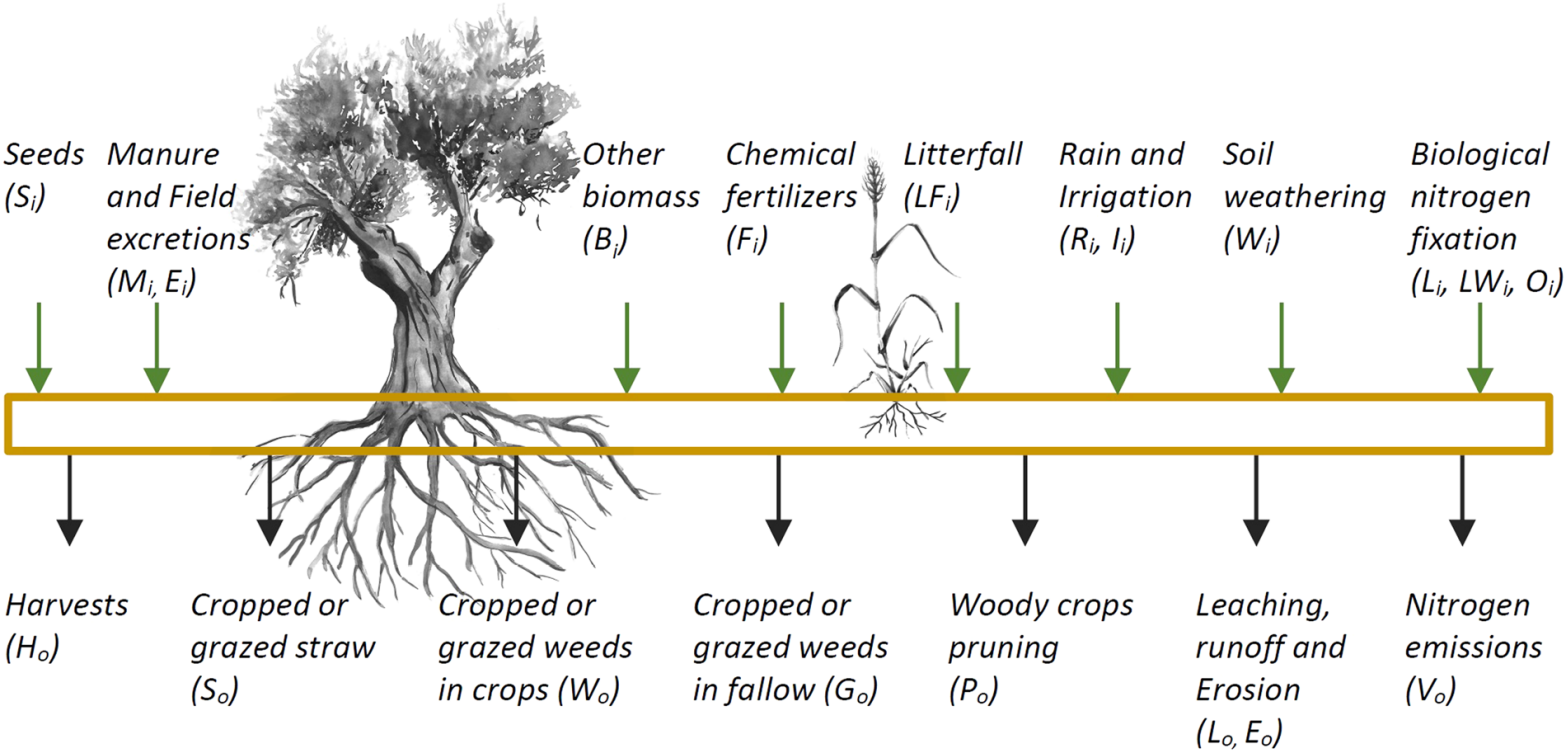
Diagram of topsoil N-P-K flows. See supplementary information file for full description of variables and model. Green arrows represent inputs and black arrows outputs. Olive tree and wheat plant drawings by Rita Hermínio.

Our work is a development of the nutrient balance methodologies established in the Agro-Ecosystems History Laboratory (Seville, Spain)^11, 32, 33^. The calculations follow a mass balance approach to the N-P-K flows of cropland topsoil, for average conditions in the period 1951–56, comprising arable crops, vegetables and woody crops (ca. 5.6 million ha) (Fig. 1). Uncultivated land such as permanent grassland was excluded. Topsoil was defined as the 30 cm upper layer of the soil, which covers most of the root activity and establishes an analytical frontier that allows a consistent modelling of soil weathering and nutrient uplift by trees. The results are amounts (kg N-P-K.yr^−1^) or surface rates (kg N-P-K.ha^−1^.yr^−1^) representing a depletion (negative) or accumulation (positive) of N-P-K in soil for a given year. See equation (1). The overall quality of the model depends on the identification of all flows and on their modeling with reliable knowledge and data. Efforts were made to include the flows associated to trees, crop weeds, soil weathering and organic fertilization from urban and marine sources. We excluded atmospheric dry changes (deposition and airborne) from gaseous and particulate transport of N-P-K due to modelling difficulties, assuming that outputs and inputs are similar and therefore the net balance is approximately zero. The gaseous exchanges of P and K were also dismissed as being minimal^34^. The complete description of the model is in the supplementary information file (SI). We present next five sets of flows selected for relevance to the results and methodological innovation.

1 $$Nutrient\,Balance\,(N,P,K)=\sum Input\,(N,P,K)-\sum Output\,(N,P,K)$$

### Study area

The area under study is the Portugal mainland (ca. 36°57′–42°09′N, 6°11′–9°30′W) with about 89,000 km^2^. Portugal has a temperate Mediterranean-type climate with Atlantic or Continental influences, where wet and cold winters alternate with hot and dry summers^35, 36^. The annual mean of average daily temperatures varied from 7.5 °C to 17.5 °C in the 1931–1960 period^37^. The mean annual rainfall for the 1950’s decade was 992 mm, ranging annually from 660 to 1470 mm (SI). Altitude rises to almost 2000 m in the Estrela mountain range with a continental mean around 240 m^37^. The soil is predominantly acid (>80% of area) except for soils resulting from carbonate or granitic alkaline rocks^37^. The northern half of the country has a hilly topography, cold and wet climate with precipitation reaching 2000 to 3000 mm annually, while the south has a plane topography with small elevations, long warm and dry summers, and a mean annual rainfall that hardly surpasses 800 mm.

### Primary data

The continental scale was preferred versus smaller regions for it increases the quantity and quality of data collected, as statistical services operated mostly at the national level. We processed data from official reports and past agronomic literature to describe land use, crops and yields, irrigation and rainfall, livestock and manure, chemical fertilization, farming practices, etc., in the study period. This data is fully provided in the SI and briefly described next.

The core data used in the model was the nationwide land use survey from 1951 to 1956, referred at that time as “the most detailed and trustable” survey ever done in Portugal^38^. Though it presents a good disaggregation of agricultural land uses it omits a fallow land class (comprised in the “arable surface” class), which we have estimated (Go flow in SI). We set different fallow land areas in the nationwide and arable crops balances, in order to avoid double counting of weeds output due to the overlap of arable crops with permanent crops (SI). Data on yield, sowing amounts and area of the arable crops (six cereals: wheat, rye, oat, barley, maize, rice; three legumes: broad bean, beans, chick-pea; and potato), vegetables, olive groves, and vineyards were available on an annual basis^38, 39^. Fruit tree data were provided by a national enquiry that counted “all the fruit trees” in the year 1954 (23 species, ca. 33.3 million trees excluding olive trees)^40^. Livestock numbers were taken from the survey of 1955 (12 species)^41^. Annual consumption of chemical N-P-K was provided by a national report of the 1940–1958 period^42^. We drew annual rainfall data from the national meteorological organization (SI) and irrigation volumes from past literature (SI).

### Fertilization inputs

We estimated the N-P-K inputs from livestock excretions combining livestock numbers with annual excretions of each species (kg.head^−1^.yr^−1^) and then converting this biomass to N-P-K. The excretions were distributed in three parts following the different species management deduced from past literature (SI): manure (Mi), field excretions while grazing either in cropland (Ei) or in uncultivated land. For example, about 50% of cattle excretions was transformed into manure, whereas 30% was excreted on cropland and 20% lost on uncultivated pastureland. For poultry, we assumed 80% as manure and distributed equally the remaining 20% between field and lost excretions. According to these calculations we estimated 7.8 million Mg of manure (which exceeds the 6 million Mg estimated by the livestock services^10^) and 5.4 million Mg of field excretions in cropland.

The N-P-K content of excretion is also species specific and was calculated by a grand mean approach over historical sources (SI). In the absence of detailed data, we assumed N-P-K content to be similar in excretions and fresh manure (although manure included straw and other plant biomass). This is valid for N and P which present similar concentrations in excretions and plants residues, but not for K: the estimated mean content of dry straw was N-0.56%, P-0.12%, K-0.63% whereas the mean manure/excretions content was N-0.54%, P-0.09%, K-0.29%. The latter are almost equal to the “farm manure” content (N-0.47%, P-0.09%, K-0.29%) published in 1898^9^ and match the N and P intervals from 52 manure analyses (N: 0.35–0.65%, P: 0.04–0.24%, K: 0.42–0.58%) carried out in France in 1956^43^. N-P-K inputs from manure (Mi) were then finalized by subtracting significant N-P-K losses due to manure management and storage before application (N-0.18%, P-0.02%, K-0.11%)^44^. The losses following field excretions and manure application were regarded otherwise as output flows (Vo). We assumed that half of manure was applied over arable crops while the other half was applied over woody crops and vegetables^8, 10^. The field excretions were distributed by crops using the area distribution of crops as proxy.

Other biomass sources were used as fertilizer during the 1950s, some traditional and other born with new industrial residues^9, 35, 45–48^. These sources are rarely included in agricultural balances^12, 15^. We considered that the most important were woodland and marine biomass, and urban waste (SI). Concerning the dynamics of mineralization and immobilization of organic matter in soil, we assumed that within a multi-year approach it is reasonable to equal the annual input of mineralized N-P-K from organic inputs to the total mineral content of that organic inputs (SI, sheet Livestock).

Mainland annual consumption of chemical N-P-K was obtained by subtracting quantities that were exported to the Atlantic Portuguese islands (ca. 4% from total) and adding organic fertilizers from industrial preparation (ca. 2%)^42^. The distribution by crop of these fertilizers was also available^49, 50^.

### Raining and weathering N-P-K

N in rainwater solution might represent a large input even in Mediterranean agroecosystems. We calculated rainfall inputs (Ri) by combining historical rainfall data and cropland area with N-P-K concentrations in rainwater (P and K show small values). The mean annual precipitation (920 mm) of 1951–56 is similar to the mean value of the 1930–2000 period^51^. For N (nitrate-NO_3_^−^ and ammonium-NH_4_^+^) and K we used averaged concentrations measured in Iberian weather stations (four in Portugal and two in Spain close to the border) for the period 1979–2010^52^. Even if N-NH_4_^+^ concentrations in the 1951–56 period are expected to be lower the biased effect should be small as suggested by the cross-checking estimates of NH_4_^+^ volatilization (Vo). The K rate is in accordance with Öborn’s range^24^. For P wet deposition, which is often estimated as null, we used the global surface rate of 0.2 kg.ha^−1^.yr^−1^ ^22^.

Soil weathering is not always considered in nutrient balances, mainly because empirical data or models are lacking, or because it is considered as a transfer from one pool (parent material) to another pool (exchangeable or soluble) within topsoil. No studies that relate lithological data with weathering rates were found for Portugal, and therefore we used N-P-K weathering rates from worldwide literature. N weathering is “conspicuously absent” from most reviews yet it may vary from trace levels in granites to ecologically significant values in some sedimentary and metasedimentary rocks (4–37 kg.ha^−1^.yr^−1^)^53^. We considered this input null (although a small relative contribution is expected) as it is difficult to deduce a reliable rate for Portugal. For P weathering, we used the global mean of 1 kg.ha^−1^.yr^−1^ ^21^, whereas the K rate was obtained from the linear function between soil clay content and measured weathering rates (R^2^ = 0.98), estimated from Öborn’s data^24^, and the mean clay content of Portuguese topsoil^54^. Certain clay soils have great ability to deliver K over long periods, even though clay influence may be contradictory: some clay minerals behave both as sources and sinks^24, 26^. The K rate was finally reduced in 58% to 7.4 kg.ha^−1^.yr^−1^, based on laboratory experiments on mineral fixation of added K^24^.

### Trees as nutrient pumps

Lehmann’s data^55^ on cumulative depth distributions of the root activity of tree crops showed that it may be vary significantly from 10% to 75% of total activity being within the top 10 cm of soil. The deepest root activity was observed for fruit trees. We selected seven tree crops from subtropical and dry tropical climate, as well the model developed for citrus and olive trees, and estimated that around 50% of the root activity of fruit trees, olive trees, and vines occurs in subsoil (below the 30 cm topsoil). Therefore, half of trees’ harvest and pruning flows (Ho, Po) came from the subsoil and were discounted from these N-P-K outputs as a bypass flow. Conversely, half of litterfall flow (LFi) came from topsoil and was discounted from this input as recirculating flow. We considered neither the nutrient uplift in the cork and holm oak savanna (ca. 1 million ha of arable crops under the Portuguese ‘montado’) nor the counterbalancing nutrient output in cork, acorn and wood because of modelling difficulties. To the best of our knowledge no prior historical nutrient balance has addressed the trees uplift input.

### Weeds outputs and inputs

Primary production of agroecosystems is partially not cultivated. A fraction of it is spontaneous flora relevant in traditional agriculture, although less relevant in modern agriculture with persistent herbicide use. The aboveground biomass of weeds, and its conversion to N-P-K, was estimated with productivity factors for historical agroecosystems in Mediterranean conditions^33^, considering that herbicide use in the 1950s was negligible^56^ and weeds ecology and dry nutrient content are similar to that of natural pastures, typified from different sources (N-1.20%, P-0.13%, K-0.93%)^57, 58^. We combined the weeds productivity for different crops (dry kg.ha^−1^) with the crops area and the mean N-P-K content of natural pastures to obtain the total amount of N-P-K in aboveground weeds.

Furthermore, we assumed that the residual biomass from cultivated fields and fallows (straw and weeds) was intensively used to feed animals and to produce manure, in the context of a generalized deficit in organic matter^10, 59^. In the case of crop weeds (Wo), we assumed that 70% of the aboveground P and K was withdrawn as an output in the same proportion assumed for crop straw (So)^60^. For N, we considered 80% to account the potential field burning practices which volatize almost all biomass N^61^. In the case of fallows (Go), the output factors were reduced to 50% for P and K, and to 55% for N (Gloria Guzmán, personal communication) (SI, sheet Weeds). The biological N fixation by legumes occurring in wild plant communities was included in the LWi input flow.

### Main losses

There are many opportunities for N-P-K to escape from agroecosystems, mainly for N^20^. Some occur before their entry into the agroecosystem and were not considered as outputs, while others take place on the topsoil via leaching and runoff (Lo), erosion (Eo), and N emission (Vo). We summarize next the output rates assumed in the model and full described in SI.

#### Leaching and runoff (Lo)

Nitrate (NO_3_^−^) is highly soluble and hardly retained in soil by geochemical mechanisms and may be lost by surface and vertical runoff (leaching). The losses were estimated as 20% of total N input in fertilizers yet the variability range is high (10% to 80%)^20, 62–65^; P shows high tendency to precipitate and the equilibrium concentration in solution is usually very low, around 0.05 mg.L^−1^ ^34, 66^. The literature does not consider P losses from leaching but from surface transport only (Eo)^15, 22, 67^. We assumed P leaching null; K leaching is expected to be small^24, 68^, but important variation has been registered (1–44 kg.ha^−1^.yr^−1^)^24^. We assumed K losses to be 2.0 kg.ha^−1^.yr^−1^ for fertilized cropland and 1.5 kg.ha^−1^.yr^−1^ for fallow land.

#### Erosion (Eo)

Since mineral N is highly soluble in water and vertical and horizontal N runoff was already considered in Lo, we assumed the surface runoff of insoluble N to be null (although the transport of organic insoluble N may occur); We estimated that 10% of total P in fertilizers inputs is lost by erosion and surface runoff^21^ that resulted nationwide in the rate of 0.7 kg.ha^−1^.yr^−1^. This rate is similar to the rate obtained for the Enxoé basin in southern Portugal and matches the intervals obtained in several basins in Navarra (Spain) and central Europe^69^; The K erosion rate (1.4 kg.ha^−1^.yr^−1^) was estimated combining soil loss rate (1200 kg.ha^−1^.yr^−1^)^70^ with mean soil K content (1132 mg.kg^−1^)^71, 72^.

#### N emissions (Vo)

We set a calculation procedure for ammonium (NH_4_^+^) volatilization based on the assumption that global emission must equal the global deposition and that the removal of emitted NH_3_ from atmosphere is fast^19, 20^ and confronted the results with the sum of plausible NH_3_ emissions from field excretions, manure, fertilizers, and basal field emissions (SI). NH_3_ emission rate was estimated as 4.1 kg.ha^−1^.yr^−1^; There is overall uncertainties on soil emissions of NO, N_2_O and N_2_ and so we decided to compute and combine different selected procedures^19, 20, 73, 74^, resulting in rates in the range 2.5–14.3 kg.ha^−1^.yr^−1^. We used a simple mean over the estimated values (5.3 kg.ha^−1^.yr^−1^). The total N gaseous losses were 9.4 kg.ha^−1^.yr^−1^.

## Results and Discussion

### Nationwide N-P-K balance

N, P and K balances for the cropland topsoil are presented in Table 1. For N we found a mean deficit of ca. 11,600 Mg.yr^−1^, i.e. −2.1 kg.ha^−1^.yr^−1^, while P and K showed a positive balance with accumulation of ca. 26,500 Mg (4.7 kg.ha^−1^.yr^−1^) and 5,808 Mg (1.0 kg.ha^−1^.yr^−1^), respectively. These aggregate sums over a cropland area of about 5.6 million ha mask differences between crops and regions but produce important outcomes nationwide. First of all, chemical fertilization was already crucial in the first half of the 1950s for the replenishment of soil nutrients. Without it soil balance would have been negative for both N and P (−7.9 kg.ha^−1^.yr^−1^ and −0.2 kg.ha^−1^.yr^−1^, respectively), although it would have remained positive for K (0.2 kg.ha^−1^.yr^−1^). Chemical N-P-K was applied to crops (excluding fallows) at mean rates of 10.9 kg N.ha^−1^.yr^−1^, 8.0 kg P.ha^−1^.yr^−1^, and 1.4 kg K.ha^−1^.yr^−1^. However, organic fertilization from livestock excretions and other sources, such as marine biomass and urban waste, represent together 34% of N, 25% of P, and 36% of K total inputs, which are greater than the chemical N and K share. In the period 1951–56 Portuguese agriculture was fertilized both chemically and organically, with biomass leading the N and K inputs and chemical compounds the P inputs. If considering only the fertilization inputs (*i.e*. Mi, Ei, Fi and Oi flows), the share of chemical fertilizers was 36% of N, 70% of P and 11% of K, with the remaining proportions coming from organic entries.

**Table 1:** The N-P-K balance for mainland Portugal cropland. The flow share is shown in percentage.

N-P-K flows (Mg)	N		P		K	
Outputs		%		%		%
Harvests (Ho)	38 828	(19.0)	6 358	(30.6)	14 192	(13.8)
Cropped or grazed straw (So)	19 081	(9.3)	3 287	(15.8)	17 918	(17.4)
Cropped or grazed weeds – crops (Wo)	42 275	(20.7)	4 004	(19.3)	28 470	(27.7)
Cropped or grazed weeds – fallow (Go)	29 514	(14.4)	2 904	(14.0)	20 650	(20.1)
Woody crops pruning (Po)	2 704	(1.3)	369	(1.8)	2 635	(2.6)
Leaching and runoff (Lo)	19 360	(9.5)	0	(0)	11 256	(11.0)
Erosion (Eo)	0	(0)	3 830	(18.5)	7 645	(7.4)
N emissions (Vo)	52 692	(25.8)	0	(0)	0	(0)
*Sum*	204 455	(100)	20 753	(100)	102 767	(100)
**Inputs**						
Seeds (Si)	3 788	(2.0)	614	(1.3)	1 167	(1.1)
Manure (Mi)	28 528	(14.8)	5 554	(11.8)	14 007	(12.9)
Field excretions (Ei)	30 357	(15.7)	4 992	(10.6)	15 438	(14.2)
Other biomass entries (Bi)	7 284	(3.8)	1 159	(2.45	9 719	(9.0)
Chemical fertilizers (Fi)	37 918	(19.7)	27 752	(58.7)	4 916	(4.5)
Litterfall (LFi)	3 275	(1.7)	280	(0.6)	2 944	(2.7)
Rainfall (Ri)	22 622	(11.7)	1 126	(2.4)	9 118	(8.4)
Irrigation (Ii)	5 569	(2.9)	152	(0.3)	9 546	(8.8)
Soil weathering (Wi)	0	(0)	5 628	(11.9)	41 719	(38.4)
Symbiotic N fixation - cultivated legumes (Li)	8 355	(4.3)	0	(0)	0	(0)
Symbiotic N fixation - wild legumes (LWi)	27 490	(14.3)	0	(0)	0	(0)
Non-symbiotic N fixation (Oi)	17 589	(9.1)	0	(0)	0	(0)
*Sum*	192 774	(100)	47 257	(100)	108 575	(100)
**Balance**						
(Mg)	−11 681		26 503		5 808	
(kg.ha^−1^.yr^−1^)	−2, 1		4, 7		1, 0	

Nonetheless, the use of chemical fertilizers was steadily increasing since 1946, and the mean consumption of chemical N in 1957 and 1958 surpassed by 50% the mean consumption of the 1951–1956 period (13% in P and 39% in K)^42^, an increase that was large enough to surpass the N gap. Even considering that this intensification resulted in increased outputs such as harvest and leaching, it is very likely that an N surplus was reached from the late 1950s onward. Additionally, our data show that chemical N input increased rapidly from 1951 to 1958 (≈118%, with annual rates ranging from 7% to 19%), finally equaling the organic entries of N (SI). The 1950s thus establish the turning point from an agriculture fertilized predominantly through biomass N, collected within or close to farms, to one where industrial N prevailed. Despite the general increase in chemical supply, K fertilization remained largely organic, while the predominance of industrial P had already been established in previous decades.

The separation between “natural” flows and those controlled by farmers is not easily set for both conceptual and methodological reasons, but remains essential to assess the passive contribution of non-human inputs to soil fertility. Natural or passive inputs (here defined as Ri, Wi, LFi, LWi and Oi) summed annually around 71,000 Mg of N (37% of total inputs), 7,000 Mg of P (15%), and 53,800 Mg of K (50%). Rainfall alone contributed with 4.0 kg N.ha^−1^.yr^−1^, 0.2 kg P.ha^−1^.yr^−1^ and 1.6 kg K.ha^−1^.yr^−1^.

Output results showed that biomass extraction through harvest, grazing and pruning was, as expectable, the greatest output (more than 80% of total P and K outputs and 65% of N). Not so obvious is the fact that the edible parts of crops account for a small fraction of nutrient uptake in biomass (N-29%, P-38%, K-17%). As reported in a world evaluation for the mid-1990s^61^, “it would not be inappropriate to define agriculture as an endeavor producing mostly inedible phytomass”. The biomass residues of the Portuguese 1950s were not anyhow the misused “valuable renewable resource” of the reported 1990’s but a key source of organic matter widely used to satisfy mutually dependent functions of past agriculture: manure, animal work, meat and household fuel. These flows represent a significant output which partly returned to soil via, mostly, livestock excretion. Lastly, the inclusion of crop weeds proved to be decisive to the balance (Table 1).

We developed a sensitivity analysis that identifies the flows whose variation has biggest impact on the balance results. We calculated the minimum (positive or negative) variation (%) in each flow that would by itself be enough to change the sign of the balance (i.e., switch from depletion to accumulation or vice-versa) (Table 2). For example, the N deficit would be canceled by the combined reduction of the outputs from weeds and gaseous emissions, and the increment of the inputs from organic and chemical fertilizations (around ± 6% in each flow). The P sign is difficult to change, mainly because the chemical input alone surpasses the sum of all outputs. For K, a reduction of 14% in the weathering rate is enough to cancel the soil accumulation obtained, which would be however reinforced by a small decrease in the weeds and straw outputs. Note that the variations of each flow have contrary effects concerning the balance of N and that of P and K, as visible by the sign variation in Table 2.

**Table 2:** The flow variation (%) that are critical for the N-P-K sign of the nationwide balance.

		N	P	K
**Outputs**	Harvests (Ho)	−**30%**	417%	41%
	Cropped or grazed straw (So)	−61%	806%	**32%**
	Cropped or grazed weeds - crops (Wo)	**−28%**	662%	**20%**
	Cropped or grazed weeds - fallow (Go)	−40%	912%	**28%**
	Woody crops pruning (Po)	(−100%)	7176%	220%
	Leaching and runoff (Lo)	−60%	—	52%
	Erosion (Eo)	—	692%	76%
	N emissions (Vo)	**−22%**	—	—
**Inputs**	Seeds (Si)	308%	(−100%)	(−100%)
	Manure (Mi)	41%	(−100%)	−41%
	Field excretions (Ei)	**38%**	(−100%)	**−38%**
	Other biomass entries (Bi)	160%	(−100%)	−60%
	Chemical fertilizers (Fi)	**31%**	−95%	(−100%)
	Litterfall (LFi)	357%	(−100%)	(−100%)
	Rainfall (Ri)	52%	(−100%)	−64%
	Irrigation (Ii)	210%	(−100%)	−61%
	Soil weathering (Wi)	—	(−100%)	**−14%**
	Symbiotic N fixation - cultivated legumes (Li)	140%	—	—
	Symbiotic N fixation - wild legumes (LWi)	42%	—	—
	Non-symbiotic N fixation (Oi)	66%	—	—

The bold values identify the smaller critical values and the (−100%) notation identified the flows with negative variation superior to the flow amount.

### The arable crops rotations

During the first half of the 1950s arable crops rotations covered over 94% (ca. 5.3 million ha) of the cropland surface, overlapping with woody crops (mostly olive groves) and cork and holm oak savanna (Portuguese ‘montado’) in about 1.7 million ha. Crop rotations were very distinct across the country and could vary considerably, even locally. In the Beja and Serpa municipalities in the south, 21 and 45 rotation types, respectively, were identified in the 1950s^75, 76^. Notwithstanding this diversity, the arable system can be observed as an abstract nationwide rotation where cereals plus potato occupied 44% of the arable land, grain legumes 10%, and fallow land 46%. The overall balance confirmed the pattern found nationwide: N presents a deficiency (−1.6 kg.ha^−1^.yr^−1^) and P and K an accumulation (4.2 kg.ha^−1^.yr^−1^ and 3.0 kg.ha^−1^.yr^−1^, respectively). The fertility meaning of the rotations only comes out when contrasting the different land uses (Table 3). For cereals and potato the N deficit reached 11,800 Mg which were not compensated by the positive balance on legume crops (ca. 2,800 Mg) and fallow (388 Mg). One legume crop left in the soil enough N (5.6 kg.ha^−1^.yr^−1^) to compensate the deficit of one cereal crop (−5.0 kg.ha^−1^.yr^−1^) while compensation by the fallow was virtually null (0.2 kg.ha^−1^.yr^−1^). N surplus in legume crops is smaller than the inputs of chemical fertilizers (14.4 kg.ha^−1^.yr^−1^) but superior to any other input in the cereal crops including manure (5.5 kg.ha^−1^.yr^−1^).

**Table 3:** The N-P-K balance for the arable crops disaggregated in cereals plus potato, legume crops and fallow land.

N-P-K flows (Mg)	Cereals and potato			Legume crops			Fallow land		
	N	P	K	N	P	K	N	P	K
**Outputs**									
Harvests (Ho)	29 343	5 134	104	4 838	549	49	0	0	0
Cropped or grazed straw (So)	13 811	2 770	13 559	1 826	219	1 886	0	0	0
Cropped or grazed weeds – crops (Wo)	19 768	1 872	13 313	4 275	405	2 879	0	0	0
Cropped or grazed weeds – fallow (Go)	0	0	0	0	0	0	33 613	3 308	23 519
Leaching and runoff (Lo)	11 888	0	4 701	722	0	1 019	3 236	0	3 654
Erosion (Eo)	0	2 832	3 193	0	118	692	0	266	3 309
N emissions (Vo)	22 009	0	0	4 768	0	0	12 642	0	0
*Sum*	96 819	12 609	34 870	16 429	1 291	6 524	49 491	3 574	30 483
**Inputs**									
Seeds (Si)	2 838	497	732	663	76	165	0	0	0
Manure (Mi)	12 837	2 499	6 303	1 426	278	700	0	0	0
Field excretions (Ei)	8 865	1 458	4 508	1 921	316	977	16 179	2 660	8 228
Other biomass entries (Bi)	2 185	348	2 916	2 185	348	2 916	0	0	0
Chemical fertilizers (Fi)	33 747	24 367	4 164	265	583	128	0	0	0
Rainfall (Ri)	9 449	470	3 808	2 047	102	825	9 793	487	3 947
Irrigation (Ii)	4 070	111	6 976	257	7	441	0	0	0
Soil weathering (Wi)	0	2 351	17 426	0	509	3 775	0	2 436	18 060
Symbiotic N fixation - cultivated legumes (Li)	0	0	0	8 131	0	0	0	0	0
Symbiotic N fixation - wild legumes (LWi)	6 326	0	0	1 368	0	0	15 646	0	0
Non-symbiotic N fixation (Oi)	4 701	0	0	1 019	0	0	8 262	0	0
*Sum*	85 019	32 100	47 101	19 283	2 218	9 985	49 880	5 584	30 724
**Balance**									
(Mg)	−11 801	19 491	12 231	2 854	928	3 461	388	2 010	241
(kg.ha^−1^.yr^−1^)	−5, 0	8, 3	5, 2	5, 6	1, 8	6, 8	0, 2	0, 8	0, 1

In the case of P and K the greater accumulation on cereals and potato fields comparing to legumes and fallow is explained by the distribution of chemical fertilizers by crops. Around 90% of chemical N-P-K was applied on arable crops, almost all on cereals and potato. Legumes received only 1 to 3% and fallows were not fertilized (SI). The removal of this input makes arable crops deficient in P (−0.5 kg.ha^−1^.yr^−1^) and the N gap grows by a factor of 5 (−8.0 kg.ha^−1^.yr^−1^).

The 1950s agroecosystem, in transition from organic to chemical inputs, reveals now a land use distribution that cannot sustain the annual balance of both N and P without chemical fertilizers. These are historically interconnected processes perceived by coeval agronomists who identified the reduction of fallow land in the first half of 1900s as a “harmful tendency”^77^ and proposed a “large scale [implementation] of lupins” to incorporate in the soil as green manure^10^. Manure was insufficient since the beginning of the 20th century^72, 78^ and in the 1950s a deficit of 25 million Mg was estimated^10^, four times the actual production of manure. Further, over the 1950s’ peak of cropland, the rapid and unprecedented spread of chemical fertilizers and mechanical strength occurred^79^. The long-standing expansion of the arable surface (and the concentration of that surface on cereal crops, mainly wheat) would not have been possible without the substitution of the “land cost” of organic farming by externalized inputs^3^ that were sufficient to balance soil P and K in the 1951–56 period and, some years later, soil N.

### Were fallows restoring fertility?

Fallow land is a nutrient replenishment practice that balances passive inputs with grazing output intensity. It is a waiting method where cropland is provisionally converted into pastureland. Nevertheless, fallow land balance was only slightly positive: N, P and K were accumulated at rates smaller than 0.9 kg.ha^−1^.yr^−1^ (Table 3). This was due to the intense extraction of biomass in fallow land by grazing that was poorly compensated by excretion on site. This is in line with the tendency for nutrient mining in grasslands, particularly in extensive agriculture^22, 24, 34^. Indeed, if grazers are removed from fallow, together with their excretions, the balance of N and K rises up to more than 6.0 kg.ha^−1^.yr^−1^ and to 1.1 kg.ha^−1^.yr^−1^ of P, providing an N surplus sufficient to overcome the N deficit of arable crops and to reduce in 1/4 the chemical N input. However, the fallow land pastures could not be dismissed in the 1950s because they were an important source of nutrients transferred to crops in the excretions. Fallows were restoring the fertility of cropland while accumulating a small or null amount of N-P-K in their soil.

Notwithstanding, fallow land could have been sown with fodder legumes following the rotations schemes developed in the 18^th^ and 19^th^ northern Europe that enhanced manure availability and N fixation^1, 80^. The cultivation of fallows was studied in Portugal for yields and fertility improvements^75, 81–83^, and was internationally recognized in 1951 by the young Organisation for European Economic Co-operation as “the outstanding problem awaiting solution in South Portugal”^82^, but it was not consensual among agronomists^84^ and their implementation was minimal at the end of the 1950s after a marginal use in the previous century^82, 85^. Further research is required to clarify the persistence of fallow land in Portugal, as well to quantify the nutrients transferred to cropland via livestock that came from grasslands nearby cropland (natural pastures, moorland, forests). This transfer is crucial to measure the total land allocated to organic farming.

The sensitivity analysis of the arable crops balance (Table 4) confirmed the robustness of P accumulation. In the case of cereals and potato, N emissions remain a critical flow. Harvest biomass and chemical fertilization emerged as the most sensitive flows to the results (−37% and 33% of critical variation, respectively). K balance appear now less vulnerable to K weathering variation comparing to the nationwide balance. The balance of legume crops is globally robust to any variation except for the symbiotic N fixation due to cultivated legumes, which is the highest flow. Conversely, the balance of fallow land, with low accumulation of N-P-K, is highly sensitive to all flows of N and K.

**Table 4:** The flow variation (%) that are critical for the N-P-K sign of the arable crops balance.

		Cereals and potato			Legume crops			Fallow land		
		N	P	K	N	P	K	N	P	K
**Outputs**	Harvests (Ho)	**−37%**	381%	11792%	61%	172%	7045%	—	—	—
	Cropped or grazed straw (So)	−80%	706%	90%	161%	432%	184%	—	—	—
	Cropped or grazed weeds - crops (Wo)	−56%	1045%	92%	69%	233%	120%	—	—	—
	Cropped or grazed weeds - fallow (Go)	—	—	—	—	—	—	**2%**	65%	**1%**
	Leaching and runoff (Lo)	−95%	—	260%	395%	—	340%	**23%**	—	**7%**
	Erosion (Eo)	—	689%	383%	—	790%	500%	—	764%	**7%**
	N emissions (Vo)	**−50%**	—	—	62%	—	—	**6%**	—	—
**Inputs**	Seeds (Si)	387%	(−100%)	(−100%)	(−100%)	(−100%)	(−100%)	—	—	—
	Manure (Mi)	86%	(−100%)	(−100%)	(−100%)	(−100%)	(−100%)	—	—	—
	Field excretions (Ei)	117%	(−100%)	(−100%)	(−100%)	(−100%)	(−100%)	**−5%**	−76%	**−3%**
	Other biomass entries (Bi)	503%	(−100%)	(−100%)	(−100%)	(−100%)	(−100%)	—	—	—
	Chemical fertilizers (Fi)	**33%**	**−80%**	(−100%)	(−100%)	(−100%)	(−100%)	—	—	—
	Rainfall (Ri)	116%	(−100%)	(−100%)	(−100%)	(−100%)	(−100%)	**−8%**	(−100%)	**−6%**
	Irrigation (Ii)	270%	(−100%)	(−100%)	(−100%)	(−100%)	(−100%)	—	—	—
	Soil weathering (Wi)	—	(−100%)	**−70%**	—	(−100%)	−92%	—	−88%	**−1%**
	Symbiotic N fixation - cultivated legumes (Li)	—	—	—	**−36%**	—	—	—	—	—
	Symbiotic N fixation - wild legumes (LWi)	174%	—	—	(−100%)	—	—	**−5%**	—	—
	Non-symbiotic N fixation (Oi)	234%	—	—	(−100%)	—	—	**−9%**	—	—

The bold values identify the smaller critical values and the (−100%) notation identified the flows with negative variation superior to the flow amount.

### The wheat case

Wheat crops occupied more than 850,000 ha during the study period, by far the largest crop corresponding alone to 30% of sown fields. This area increased gradually from less than 300,000 ha in the late 19^th^ century^86^ and inflected in the last years of the 1950s until the present, where the wheat area hardly exceeds 50,000 hectares^31, 38^, in an evolution strongly set by political and economic drivers^27, 87, 88^. That large surface from 1951–56 was concentrated (80%) in the southern half of the country. Wheat is a major exporter of N-P-K in grain (Table 5) and, not surprisingly, shows an N deficit (−5.9 kg.ha^−1^.yr^−1^, Table 6) higher than that of cereals and potato taken together. The higher accumulation of P (13.2 kg.ha^−1^.yr^−1^) but not of K (0.7 kg.ha^−1^.yr^−1^) is explained by the distribution of chemical fertilizers (SI). Chemical P was mainly applied on wheat (around 50% of national consumption) whereas K fertilization was concentrated on potato and maize crops (only 12% on wheat). The proportion of chemical N applied on wheat (32%) was similar to its area proportion in the sown fields. The apparent mismatch between fertilization and the needs indicated by the wheat balance (also observed in the arable crops), is partly explained by the difficulty in accessing N fertilizers until the end of WWII and by mistaken cultural practices that promoted exclusive P fertilization in extensive farming since the late 19th century^89–91^.

**Table 5:** The grain N-P-K output rates (kg.ha^−1^.yr^−1^).

	N	P	K
**Wheat**	**15.0**	**2.6**	**3.3**
Rye	10.6	2.2	2.7
Oat	6.7	0.8	1.2
Barley	8.4	1.7	2.2
Maize	13.0	2.0	2.2
Rice	40.7	10.0	8.4

**Table 6:** The N-P-K balance for wheat crops.

N-P-K flows (Mg)	N	P	K
**Outputs**			
Harvests (Ho)	12 843	2 213	2 819
Cropped or grazed straw (So)	5 917	1 084	4 386
Cropped or grazed weeds - crops (Wo)	7 194	681	4 845
Leaching and runoff (Lo)	4 845	0	1 714
Erosion (Eo)	0	1 541	1 164
N emissions (Vo)	8 023	0	0
*Sum*	38 822	5 519	14 928
**Inputs**			
Seeds (Si)	1 376	237	302
Manure (Mi)	8 558	1 666	4 202
Field excretions (Ei)	3 232	531	1 644
Other biomass entries (Bi)	728	116	972
Chemical fertilizers (Fi)	12 437	13 210	590
Rainfall (Ri)	3 445	171	1 388
Soil weathering (Wi)	0	857	6 352
Symbiotic N fixation - wild legumes (LWi)	2 302	0	0
Non-symbiotic N fixation (Oi)	1 714	0	0
*Sum*	33 792	16 789	15 548
**Balance**			
(Mg)	−5 030	11 270	620
(kg.ha^−1^.yr^−1^)	−5,9	13,2	0,7

The wheat N imbalance could have been rectified by increasing chemical fertilization from the actual 14.5 kg.ha^−1^.yr^−1^ to 20 kg.ha^−1^.yr^−1^, which arose in the turn to the 1960s^42^. Alternatively, the rectification could have been achieved by increasing the area of legume crops. On average legumes left in the soil 5.6 kg.ha^−1^.yr^−1^ (Table 3) while wheat depleted the soil at 5.9 kg.ha^−1^.yr^−1^ (Table 6). Therefore, legumes surface should equal, approximately, that of wheat (1:1). This would have represented an increase in legume area from 500,271 ha to 856,959 ha (>68%), which would be achieved with biennial wheat-legume or triennial wheat-fallow-legume rotations. Anyhow, if the chemical fertilizers are completely excluded, the range of options for nutrient replenishment narrows. The area ratio wheat-legume goes to 1:4, only achievable, considering that cropland area in the 1950s could not grow more^59, 92^, with a significant reduction of cereals area and/or through fallow cultivation.

The expansion of nutrient demanding crops such as wheat against fallow land and other crops, that are not so demanding or even enrich the soil, depended on the increased use of chemical fertilizers, which were insufficient to balance soil N during the study period and most likely in the previous decades, since mean chemical N use in the 1940s equals 1/3 that of 1951–56^42^.

The sensitivity analysis of wheat balance (Table 7) confirmed again the robustness of P accumulation. In the case of N, the harvest outputs and the inputs from manure and chemical fertilizers controlled the main sensitivity of the results. K balance is quite sensitive to most flows, especially the weeds output and the manure and weathering inputs, due to the low positive balance. We observe also the apparent redundancy of chemical K in contrast to chemical P or N.

**Table 7:** The flow variation (%) that are critical for the N-P-K sign of the wheat balance.

		N	P	K
**Outputs**	Harvests (Ho)	**−38%**	511%	22%
	Cropped or grazed straw (So)	−82%	1042%	**14%**
	Cropped or grazed weeds - crops (Wo)	−68%	1658%	**13%**
	Leaching and runoff (Lo)	−99,8%	—	36%
	Erosion (Eo)	—	732%	53%
	N emissions (Vo)	−61%	—	—
**Inputs**	Seeds (Si)	354%	(−100%)	(−100%)
	Manure (Mi)	**57%**	(−100%)	**−15%**
	Field excretions (Ei)	142%	(−100%)	−36%
	Other biomass entries (Bi)	669%	(−100%)	−64%
	Chemical fertilizers (Fi)	**39%**	−86%	(−100%)
	Rainfall (Ri)	142%	(−100%)	−45%
	Soil weathering (Wi)	—	(−100%)	**−10%**
	Symbiotic N fixation - wild legumes (LWi)	212%	—	—
	Non-symbiotic N fixation (Oi)	285%	—	—

The bold values identify the smaller critical values and the (−100%) notation identified the flows with negative variation superior to the flow amount.

### Woody crops: orchards, olive groves and vines

Numerous studies from the last two decades on tree and shrub vertical transport of nutrients indicate an overall improvement of the nutrient status in topsoil close to trees and shrubs^55, 93–97^. Studies on Iberian *Quercus sp*. ‘montado’ showed a horizontal gradient of topsoil nutrient content related to the distance from the tree trunk^98–101^. Moreover, tree roots may also increase soil organic matter, reduce leaching, and improve soil physical properties^102^. Although these vertical and horizontal patterns present significant agroecological complexity, also influenced by the behaviour of herbivores and tillage practices, the action of trees has been proposed as the determining factor^93^. Nutrients absorbed by roots in the subsoil layers are transported within the plant and released via leaf and fruit abscission over the topsoil. There is also direct leaching from leaves by throughfall^97^ and vertical hydraulic redistribution from deep roots to superficial ones^103^.

The area of woody crops was more than 1.1 million ha and about half included cultivated fields (ca. 0.6 million ha). However, their nutrient balance cannot be performed with accuracy due to the overlap with arable crops and lack of detailed data. We do not know which crops and rotations were mixed with trees, nor the fertilization and grazing pattern of these cases. Nevertheless, nationwide results showed that woody crops contributed with a net input of N-P-K gathered in subsoil and deposited as litterfall. Further, the uplift movement of nutrients from subsoil to topsoil operated by trees represent, in addition to reduced outputs in fruits and prunings, an overall improvement of soil properties and mitigation of leaching, which may be relevant to Mediterranean agriculture as suggested by the literature. The clarification of the advantageous conditions of herbaceous and arboreal layers overlap requires building nutrient budgets for specific combinations of arable crop rotations and woody crops.

### N-P-K overall analysis

N was by far the nutrient with larger flow in the agroecosystem topsoil, as well the element with the greatest number of input and output routes. Moreover, N shortage is pointed out as the most common reason for low yields^5^. Nationwide, about 400,000 Mg of N entered and exited annually the cropland soil versus around 68,000 Mg of P and 211,000 of K. Its high solubility and reactiveness explain the high flows of some outputs (leaching and gaseous emission) and inputs (rainfall and irrigation) by comparison with P and K. N losses equal about 70% of fertilization entries, which seems acceptable in southern Europe^5^. This resulted from the careful assessment of leaching, runoff, and gaseous emission, yet general uncertainties persist in the biogeochemical cycle of N^19, 104^. N was also the larger nutrient in harvest (except for potato) and in livestock excretions, although it tends to equal K in straw, grass, wood, fruits and vegetables. Importantly, atmospheric N fixation by rhizobium associations with legumes roots and other non-symbiotic organisms contributed together with 9.6 kg.ha^−1^.yr^−1^ to the 1951–56 cropland.

The N deficit found with different breakdowns indicates, considering its biogeochemical characteristics, a consistent loss in the soil pool of plant-available N. Some farms, crop rotations or regions may have succeeded in balancing or even increasing N levels but the overall results convincingly show that Portuguese agriculture lacked N in the early 1950s. We did not consider the erosion output of insoluble soil organic N, which would have incremented N deficiency and thus does not affect the overall results and implications. Conversely, the input of N by weathering may have reduced the deficit in some specific lithologies.

P had the smallest nutrient flow. It presents relatively low requirement by plants biomass, leaching was practically absent, and wet inputs summed 0.2 kg.ha^−1^.yr^−1^. Total nationwide losses were around 10% of fertilization entries. Weathering tend to surpass losses but a large proportion of plant-available P released by weathering becomes again unavailable due to precipitation or adsorption, though generally in forms with larger specific area that are more susceptible to renewed weathering^34^. This dynamic between available and immobilized P together with small losses, which typify also K cations, suggests that the positive balances observed in all P analysis express an effective increase in the plant-available pool during 1951–56. This conclusion is in accordance with Whitehead^34^ who observed in P fertilized grasslands a consistent increase of total P in topsoil and a progressive reduction of yield response to fertilization. This trend was likely maintained in the following years by the intensification of chemical fertilization. The contribution of chemical P to inputs was very high (58.7%), slightly surpassing the soil surplus in the study period. This indicates the opportunity for improvement of P efficiency use, but also the potential underestimation of P losses in the model. Finally, considering (1) the small losses and small natural entries of P, as well (2) the similarity between harvest outputs and organic fertilization inputs, it seems that the surplus of P could have been achieved in the absence of chemical P by increasing both organic fertilization and it sources from outside the cropland (permanent pastures, urban waste, marine biomass).

K presents somehow an intermediate situation. The magnitude of K circulation in biomass was high (ca. 127,000 Mg versus 205,000 Mg of N and 29,000 Mg of P) and losses were already substantial (3.4 kg.ha^−1^.yr^−1^), equaling 44% of fertilization inputs. The combination of the absence of gaseous losses, the high content in crop residues and weeds (also in Smil^61^) and the low retention in herbivores^34^ indicates a high opportunity for K recycling in systems that combine crops and grazing. This partially explains the reduced use of chemical K (less than 1/5 of chemical N or P). Other reasons might be the high weathering and the complex dynamic between K soil fractions that tend to rapidly equilibrate the depletion of soluble K^24^.

The soil accumulation of K obtained in the different balances is large enough to cope with the reduction of the weathering estimates (from 14% to 41%) or the total exclusion of chemical K input, which suggests an effective K surplus in the early 1950s, probably increasing both the plant-available pool and losses. This is a general overview since K weathering is strongly soil-type dependent and K deficits may have occurred in some specific crops such as potato and maize, where chemical K was concentrated.

## Conclusions

The N-P-K balance of Portuguese cropland topsoil in the 1951–56 period showed a consistent deficit in N, both in the nationwide and arable crops assessments. N deficiency was probably also present in the preceding decades. Anyway, the increment of chemical N use in the following years (1957–58), in the context of a continuous growth since the end of WWII, was enough to fill that gap. N deficiency appears to be at the center of the soil degradation described and widely recognized by numerous agronomists in the 1930–1960 period^59, 77, 105, 106^ and by more recent authors^31, 92^, as the outcome of historical relations that included the expansion of the agricultural frontier and wheat surface, limited N fertilization and fixation, progressive soil depletion and increased erosion. In contrast, P and K presented significant accumulation during the 1951–56 years that must have resulted in increased soil reserves and losses that were likely kept up or reinforced in the following years.

The early 1950s balances together with the evolution of chemical fertilization consumption provide a snapshot of the inflection from an agriculture fertilized predominantly through recycled N in biomass to one where chemical N prevailed. Chemical K and P supply was also growing (though not as much as N), but K fertilization remained mostly organic whereas chemical P predominance had already been set in previous periods. This intensification occurred when the country cropland reached a record high, never before achieved or repeated. Portugal missed the agricultural ‘revolution’ of fallow replacement by fodder crops, going directly to the ‘revolution’ of chemical fertilization (and motorization). This pattern, both distances and brings closer Portugal’s agricultural history to that of northern Europe, namely with respect to the United Kingdom where N fixation by legumes, a long used technology, peaked around 1950 and was rapidly replaced after WWII by industrial fertilizers^80^. This reinforces the idea of a post-1950s European convergence^107^ and acceleration^4, 108, 109^.

The transition towards chemical fertilization enabled and promoted the dissociation between livestock and plant production observed from the 1950s onward, nowadays fully developed. For livestock, the progressive substitution of pastures by imported feed stuffs during the 1953–1989 period was also verified^92^. This dissociation, together with the important land abandonment from the 1960s onward, is pointed out by several authors as the core process of contemporary environmental problems related to land use, such as the degradation of soil and water by intensified agriculture and husbandry (nitrate leaching, pesticides, livestock effluents, erosion, etc.)^31, 92^, on the one hand, and the growing vulnerability of the territory to wildfires, on the other hand. Wildfire occurrence in Portugal has risen in the last few decades resulting from fuel accumulation linked to the abandonment of agricultural fields and shrublands used as pastures, as well to the increase of planted forests (mainly *Pinus pinaster* and *Eucalyptus globulus*)^48, 110^.

Finally, the adaptation of arable crops towards a larger integration of legumes would have improve N availability. Additionally, several cities were adapting their waste and sewage systems to produce organic fertilizers during the 1950s (SI). The great loss of nutrients related to it was criticized since at least 1875^111^ but the advances succeeded in the mid-20^th^ century were either abandoned or remained limited. This stresses the need to unfold the different technical and social pathways that were faced during the 20^th^ century transformations, as proposed by recent histories of technology and social ecological change^2, 112, 113^ and also explored in past rural Portugal^30, 79^, as a way to critically evaluate past and present possibilities for agroecosystems.

### Data Availability Statement

All data generated or analysed during this study are included in this published article (and its Supplementary Information file).

## Electronic supplementary material

SI
